# CBSI as a Social Innovation to Promote the Health of Older People in Japan

**DOI:** 10.3390/ijerph18094970

**Published:** 2021-05-07

**Authors:** Saori Yasumoto, Yasuyuki Gondo

**Affiliations:** Graduate School of Human Sciences, Osaka University, 1-2 Yamadaoaka Suita-shi, Osaka 565-0871, Japan; ygondo@hus.osaka-u.ac.jp

**Keywords:** social innovation, community-based social innovation, health promotion, population aging

## Abstract

In this paper, we introduce a concept called community-based social innovation (CBSI). CBSI programs have been introduced to improve the physical and psychological well-being of older people. CBSI programs encourage older people to (1) take care of themselves and their peers, (2) sustain their well-being, and (3) create a cohesive and inclusive community. Although the emergence of CBSI programs is a global phenomenon, the variations, effectiveness, and sustainability of these programs are unknown. To uncover information about the Japanese version of CBSI programs, we conducted observations and face-to-face interviews with related personnel at two CBSI programs in rural areas of Japan in 2018. We found both positive and negative aspects in the current form of CBSI programs. As for the positives, these programs promote older people’s physical and psychological well-being and enhance community cohesiveness. However, CBSI programs face challenges, including that groups tend to be gender and age specific: men and the younger-old are less likely to show interest. A group-specific approach to CBSI programs could cause future community division, which would be contrary to the goal. Given the continued advancement of the aging population, a new approach to participant recruitment is needed.

## 1. Introduction

In 2019, 1 in 11 people in the world were recorded as being over 65 years old. That is equal to 9% of the world population. Especially in Europe and North America, population aging is accelerating, and one out of four people will be over 65 years old by 2050 [1]. In Japan’s case, one out of three will be over 65 years old by 2050 [2]. A worldwide phenomenon of population aging forces us to face many challenges. On the social level, for example, the decreasing size of the labor force restricts a nation’s economic growth [3]. Covering medical expenses for older people has become a national concern [4]. On the personal level, many older people who used to be taken care of are now expected to provide care for other older people (e.g., parents, spouse, siblings) [5,6,7], and people of various ages worry about how they will financially support themselves during their extended lifespan after retirement [8,9,10]. Although each country faces specific challenges depending on its cultural and social background, many countries share the need to create new forms of health and social care systems to sufficiently support the growing number of older people. The creation of a system in which older people take the initiative to guide their peers in the community through healthy aging is proposed. One such example is community-based social innovation (CBSI) [11].

CBSI programs try to improve older people’s physical and psychological well-being. More specifically, CBSI programs have been established to encourage older people to (1) take care of themselves and their peers, (2) sustain their physical and psychological well-being, and (3) create a cohesive and inclusive community [12]. WHO suggested a definition of CBSI programs based on observational research focused on low-income countries [11]. A systematic literature review related to CBSI programs in middle- and high-income countries revealed that these programs directly impact older people’s physical and psychological health. The programs also function to indirectly enhance older people’s well-being by increasing social interaction and motivation [11]. Although the accumulation of knowledge about CBSI programs across the globe is growing, drawbacks exist. For example, we know that older individuals benefit from participating in these programs; however, we are unsure whether programs are cost-effective from the government’s perspective. The extant literature also does not mention the issue of the programs’ sustainability [13].

In this paper, we introduce the emergence, development, and challenges of Japan’s CBSI programs by showing two case programs located in northern Hyogo Prefecture. We selected them as our case sites because depopulation is advancing in that area, and older people have limited interaction with younger people, while commercialized care facilities are seeking support. Thus, communities pay great attention to a system that focuses on older people’s self-support and community cohesiveness. By examining these two CBSI programs located in rural areas, where the influence of population aging is especially striking, we discuss how they function in the community and potential ways they can improve.

Japan is the leading country concerning the expansion of an aging population and has accumulated CBSI program experience. As many countries will follow what is happening in Japan to a certain extent, this case can be a model, whether good or bad. We analyzed our case CBSI programs focusing on four aspects: (1) What is the appropriate timing/social context to promote the CBSI programs? (2) What are the power relationship dynamics among the people involved? (3) What are the benefits and drawbacks of CBSI programs for people and society? (4) Would a financial investment assist in the success of CBSI programs? These four aspects are efficient for improving the current form of CBSI programs, including cost-effectiveness and sustainability [14].

### 1.1. Local Comprehensive Care System in Japan’s Super-Aged Society

Japan’s aging population is well-known in the world. Briefly, Japan became an aging society (in which 7% of the entire population is 65 years of age or older) in 1970, an aged society (14% of the population) in 1994, and a super-aged society (21%) in 2007 [15]. The rate of population aging became 27.7% in 2017 [16], and this is expected to increase to 30% by 2025 [15]. The year 2025 is especially crucial, because eight million of Japan’s baby boomers, born between 1947 and 1949, will become old-old (75–85 years old). At that point, the current social system will no longer support the elderly [16]. As population aging has advanced in Japan, the social security system has been amended multiple times. For example, elderly care used to be a family matter, and three-generation households were typical. The younger generation used to care for their older family members under the same roof; however, this began to change drastically after World War II [2]. Due to urbanization, younger people migrated to cities to seek job opportunities; consequently, older people in rural areas were left behind [17,18,19]. Even in urban areas, the individualization of the family, a concept introduced from Western countries, became internalized in people’s lifestyles [20,21]. Consequently, one-generation households became more common than three-generation households: approximately 50% of families formed three-generation households in 1986, whereas this figure was 12.2% in 2015. Moreover, nearly 60% of people over 65 years of age lived in either a single household or a couple-only household in 2015 [15].

To respond to these changes, the Japanese government enacted the long-term care insurance system in 2000. This system’s motto was “From care by a family to care by society”, under which people over 65 are entitled to receive long-term care based on their physical condition. To become a beneficiary, an elder’s health is evaluated based on a nationally standardized needs-certification system. Despite this policy, the Japanese government began to intervene in elder care responsibility [22]. The policy was said to be both effective and ineffective. One group of researchers argued that the number of older people who used public nursing care services increased, and family members were somewhat released from the burden of care. However, others criticized the policy for being inefficiently used [22,23]. Meanwhile, because of the acceleration of population aging, state financial and personnel resources have been used up. The government had to find a breakthrough idea; therefore, the long-term care insurance system was revised to become the local comprehensive care system in 2005. The main goal is to create a system in which older people can receive necessary medical and nursing care in their local community. Local comprehensive care centers were established in many cities throughout Japan, and they became responsible for reconstructing local infrastructure such as hospitals and nursing homes. The specialized position of care manager was created to assist with clear communication among older people, social workers, public health nurses, doctors, nursing home staff, etc. The care manager’s role was vital, because the lack of communication among care providers had created a situation in which some older people were unable to receive the necessary treatment. By having better infrastructure and social network systems centered around care managers, older people can quickly access the necessary care [24].

### 1.2. CBSI Programs under the Local Comprehensive Care System

The expansion of healthy longevity is a vital feature with regard to older people in Japan. For example, the Cabinet Office [2] reported that longevity had increased by almost 7 months for men, and approximately 5 months for women between 2013 and 2016. Additionally, healthy longevity increased by 9.5 months for men, and approximately 6 months for women. The increase in healthy longevity is more significant than that of longevity in general, which means that older Japanese people can live a comparatively longer healthy life before they lose their health and their lives come to an end. Although the increase in healthy longevity may help to reduce national medical expenses for older people, rapid population aging has a more significant effect.

One approach to reduce the national medical expenses incorporated in the local comprehensive care system is to encourage older people to avoid making medical and nursing care necessary by taking care of their health as long as they can. Through this approach, the Japanese government aims to expand the duration of healthy longevity to reduce the number of years when older people would have to rely on the national medical insurance system. The Japanese government suggested starting health promotion programs run by older people using traditional shared territorial bonds that already exist [24]. In 2014, public nurses from the local comprehensive care centers began explaining to residents about the situation with current long-term care insurance and population aging. They also discussed the importance of the health promotion program based on self-help and mutual help among older people in the community. For the health promotion program to be effective, exercise must be done regularly; therefore, group units were based on neighbors who could walk together to the meeting place. Groups of people who showed a willingness to start the program received financial support to renovate the meeting place (such as replacing the floor or enabling barrier-free access) and purchasing weights for physical exercise. Public nurses were scheduled to visit the groups within the first 3 months to teach appropriate exercise methods.

The health promotion programs began in 2015. The primary goal was to provide a chance for older people to practice appropriate exercises to sustain their physical health in order to live independently. Older participants were also expected to enhance their psychological well-being by conversing and exchanging support with other participants. Additionally, older people gained social roles by participating in health promotion programs. Ultimately, communities were able to develop social capital and cohesiveness. Thus, the health promotion program for older people under the local comprehensive care system contains the characteristics of CBSI programs, which are: (1) a spirit of self-help and mutual help, (2) promotion of physical and psychological health, and (3) community cohesion [13].

### 1.3. Types of CBSI across the World

The World Health Organization (WHO) Centre for Health Development was established in Kobe, Japan, in 1995 by the executive board as a global research center. Known as the WHO Kobe Centre, it is a part of the organization’s headquarters. The WHO Kobe Centre initiated research to understand variations of CBSI programs worldwide, focusing on low- and middle-income countries [25]. Based on 10 case studies, the researchers pointed out a few similarities. For example, older people who participate in CBSI programs are encouraged to enhance their psychological well-being and sense of community. CBSI participants also create receptive environments. Regarding the drawbacks, an appropriate assessment approach for CBSI programs has not been developed, thus there is insufficient knowledge about the long-term influence of the programs on participants’ physical and psychological health benefits. Additionally, many CBSI programs are run at low cost, which makes expanding them challenging. Although these programs tend to be part of the national policy on older people, program members have restricted interaction with local health and social care providers, so a direct impact on policy change cannot be made [25].

By analyzing the details of 10 case studies, WHO Kobe Centre created categories of CBSI programs. The first type of program is foundational. The scale of this type is small, and in such a program, older people who need help provide support for each other. Participants gain limited empowerment to influence the policy on older people. Case studies conducted in China and Serbia were identified as being this type. User-driven is the second type of CBSI, such as in Ukraine, Lebanon, and Thailand. Members of user-driven programs tend to be upper-class women. They often run educational and/or training sessions at the local university. Membership fees and personal funds are the main sources for sustaining these programs. Although participants can gain empowerment to influence the programs, men and working-class women have little access [25].

The third type is state-supported. This type of program is run with a top-down approach by the national government. Members may not have much power to influence the function of their programs; however, older people who need immediate support are likely to be recruited. CBSI programs examined in Russia and Vietnam used this form. The last type of CBSI program is adaptive. Participants can take the initiative and run the program while having connections with local and national systems. The adaptive type of CBSI program allows recruitment of a large number of people, but it is costly. Chile, Sri Lanka, and Iran were reported to utilize this type of CBSI program [25]. There are two significant differences between Japan and the countries mentioned above: Japan is a high-income country and has an exceptionally fast aging rate, together with an increasing percentage of old-old and oldest-old. Thus, the WHO Kobe Centre encouraged us to identify how Japan’s CBSI programs function. Additionally, the Japanese government has modified policies targeted at older people multiple times due to the rapid advancement of population aging; thus, approaches to developing CBSI programs are unique in the country.

## 2. Materials and Methods

### 2.1. Case Sites

To research the effectiveness and sustainability of CBSI programs in Japan, we conducted two field studies. The two CBSI programs are in northern Hyogo Prefecture, facing the Sea of Japan, but also surrounded by mountains. Due to their natural richness, fishing, agriculture, and forestry used to be thriving industries in this area, but their survival is in crisis because of severe depopulation and population aging. We decided to observe CBSI programs in these areas for the following reasons. In urban areas, health promotion programs based on commercial programs are becoming popular. Various programs are available, and older individuals can choose a program depending on their physical strength, interests, and schedule.

Conversely, commercial health promotion programs are not popular in rural areas. According to the Ministry of Internal Affairs and Communication [26], there are 5.26 fitness clubs per 100,000 people available in Tokyo compared with 3.57 in Hyogo Prefecture. Rural residents, therefore, have reduced access to fitness clubs. Moreover, older people in rural areas are less likely to feel that it is necessary to engage in purposeful physical exercise because they regularly work in fields. Additionally, because older people were used to receiving care prevention services from the local government under the previous policy, they were reluctant to start a health promotion program independently. Therefore, the local government’s initiative and support were necessary to establish and manage CBSI programs, even though they were expected to be run by and for local older people. Additionally, transportation systems such as buses and trains are underdeveloped in rural areas; therefore, visiting a health promotion program center can be burdensome for older people unless it is close enough to walk. Therefore, a social innovation such as a CBSI program plays an important role, especially in areas away from large cities.

We note that older people who live in areas of our case sites have other gathering opportunities and formal/informal care services supported by the local government. For example, women gather to practice Japanese kimono dressing, flower arrangement, and cooking. Older people also have access to health and osteoporosis check-ups provided by the local government. Besides, nursing care centers, day-care centers, short-stay services, and home-visit care are available depending on the older individual’s health conditions. These support systems are different from CBSI programs because they are not community-based. In most cases, participants in CBSI programs are healthy enough not to receive formal services.

### 2.2. Case 1 CBSI

Case 1 CBSI started in 2014. Following the policy, public nurses from the local comprehensive care center gathered local older people. They explained that the current elderly care insurance system would change and the insurance coverage would be reduced. The public nurses also discussed the area’s depopulation and population aging, and stressed the importance of taking care of one’s health. Health promotion programs based on self-help and mutual help (CBSI) were introduced, and older people were given time to think about their willingness to start on a program. As CBSI programs under the local comprehensive care system rely on older people’s initiative, 2014 was a significant turning point in changing their mindset and forming communities. Case 1 was one of the local communities that showed a willingness to start a program.

The introduction of this CBSI program was particularly meaningful in this community. When the long-term care insurance system was enacted, older people who were certified as needing care began to go to day-care centers. The system supported older people and their family members, but it caused an unexpected consequence for the community. The older people who went to day-care centers were labeled as weak and dependent, even though they may have been able to carry out daily routines independently. They also had fewer opportunities to share time and space with healthier older people in the community. The classification of older people based on their health conditions separated the community and removed the sense of community. To recover from such a situation, CBSI programs based on self-help and mutual help were particularly necessary to regain the sense of community.

Another essential feature of Case 1 was the presence of a human resource center for older people in the community. This center was registered as a public interest incorporated association. People over 60 years of age who are willing to work, whether paid or unpaid, can become members and work to provide services for the community’s various needs (e.g., mowing, snow shoveling, and childcare support). The CBSI program was generally run by a group leader and older participants with a public nurse; however, a few staff members from the human resource center were added to motivate the community in Case 1. The basic formation of Case 1 is that a public nurse teaches human resource center staff members how to conduct physical exercises with older people. For the first 3 months, the public nurse and staff members visited every meeting of Case 1 to teach older participants how to conduct safe and effective physical exercise. After 3 months, their visits became less frequent to check on the progress. As the CBSI programs aim to help participants maintain physical strength and psychological happiness by doing light physical exercise with peers, they cannot be replaced with professional care.

Participants of Case 1 met once a month at the public hall, which was renovated for this purpose. According to the leader, almost all participants regularly attended the meetings. They tried to meet biweekly in the past, but it was too tiring to handle, thus the participation rate declined. To make it feel fun and comfortable, they decided to meet once a month. The meeting place was barrier-free, with wood floors, so that older people could exercise safely. When we arrived at the meeting, the leader welcomed us and told us the area’s history and his group’s activities. A few supporters came before other participants arrived to prepare the activities. On the day of our visit, 3 men and 19 women participated in the program, doing physical exercise and cognitive games and writing in a nutrition diary. According to the leader, the participants’ average age was 80 years.

### 2.3. Case 2 CBSI

Case 2 CBSI also started in 2014. The advancement of population aging and depopulation is a serious concern where Case 2 CBSI operates. The town was designated as one that has the potential to disappear in 2014. This group showed their willingness to start the health promotion program soon after receiving the public nurse’s explanation. Before starting the program, participants invested in preparing the public hall for physical exercise. Many members of Case 2 were already doing volunteer work, tea parties, and short trips as a group; therefore, people within the group already had roles (e.g., leader or assistant). However, they ensured that the CBSI program remained separate from other activities. When the members met as the CBSI program, they always conducted physical exercise, thus some members only attended the CBSI meetings or the other activities.

In Case 2, the public nurse visited every group meeting during the first 3 months to teach safe and efficient physical exercise methods. At the first meeting, the participants measured their physical abilities (e.g., grip strength and standing-up motion); they then measured these every 3 months to assess the influence of physical exercise. As in Case 1, the leader and participants of Case 2 learned basic knowledge about how to conduct physical exercise during these 3 months. Through the lesson, they learned how to modify their exercise if participants had injured knees or wrists.

The Case 2 group meets every other week, but they have to take a break during the wintertime because of the snow. The day we visited, they conducted physical exercise by using weights on their wrists and ankles, singing with a sign language accompaniment, and playing cognitive games. The meeting lasted approximately 90 min. Of the 18 participants, 2 were male and 16 were female. Like Case 1, almost all the members were regular participants, so that people used the meeting to check on each other’s health conditions. The average age of participants, according to the leader, was 80 years old.

### 2.4. Data Collection and Data Analysis

We conducted participant observation and interviews from October through December 2018. For the field observation, we visited the biweekly/monthly CBSI gatherings. Before the participant observation, we conducted 90-min interviews with the related personnel (care managers and public health nurses). The interview took the form of a one-on-one meeting or a focus group (see Table 1 for the list of interviewees). We interviewed leaders of CBSI programs, but older participants were shy and expressed unease at being formally interviewed; therefore, we casually asked about their experience with monthly meetings and the evaluations after their activities were over. We tape-recorded the interviews with the permission of interviewees and transcribed all interview data (see Supplementary Materials for the interview questions). We arrived at each site 30 min before the activities began to observe their preparation process setting up the venue and greeting each other. As we had prior communication with and consent from care managers and public health nurses about the purpose of our visit for the interviews and observations, the schedules for the day went smoothly.

## 3. Results

As we described above, Case 1 and Case 2 CBSI groups manage their activities based on shared conditions and the specific features of each community. This section describes how social innovations such as CBSI programs in Japan can be improved by answering four major questions [14].

### 3.1. What Is the Appropriate Timing/Social Context to Promote a Target Social Innovation?

Answers to this question depend on the perspectives of related personnel. From the government’s perspective, population aging and a tight national budget triggered the establishment of CBSI programs. When the local comprehensive care system was introduced, the government tried to promote self-help among older people and mutual help among neighbors based on help from public services. However, the provision of public service help reached its limit, and the government needed to strengthen aspects of self-help and mutual help for older people in the community.

From the perspective of local communities, the system of care for older people needed to be renewed. Interviewee B from Case 1 CBSI said, “Under the previous policy, older people who are about to need nursing care were given a ride to participate in the care prevention program. People did not prefer such an approach, and the policy was unsuccessful”. Older people who were encouraged to participate in the care prevention program were those who could still carry out daily routines, such as taking a bath and having meals by themselves, but they needed light assistance with cleaning the bathtub and cooking, for example. They could improve or sustain their physical strength by doing exercise regularly, but might lose these abilities otherwise. Interviewee A agreed with this. Interviewees I, J, and K from Case 2 similarly talked about the previous policy. Two main reasons for the unpopularity of the previous policy emerged from their narratives. First, the local government tried to recruit participants from already running care prevention programs, but the attendees were healthy enough, therefore “they were not the right target”. Second, the local government sent out invitation letters to residents to recruit more participants. The letter included a health checklist to identify those with a high risk of frailty [27]. While this initially seemed a promising approach for recruiting participants, older people in the community felt uncomfortable participating because “it symbolizes unhealthy and dependent”.

Consequently, the participation rate was low. The collectivist culture can be complicated. On the one hand, people are willing to help each other to achieve a group goal without mentioning dissimilarities in personal characteristics, such as one’s health condition; however, it can become an issue when one’s differences challenge the harmony of the group. Group members may see that the person is causing trouble. Even if the group members do not perceive it this way, the person may feel that they are becoming a burden to the group.

The local government was also aware that their traditional assistance program for older people, called a salon (like a book club), was inefficient. A group of older people organized a salon based on a traditional shared territorial bond. The salon members were mainly women, and they used this time to share conversation. It did provide comfort for the female participants. When asked why men did not join it, Interviewee L from Case 2 said, “Men do not care about chatting”. At the same time, one of the female CBSI participants who also attend a salon told us that they do not even announce the salon for men in their community. Although a salon has a positive function for female participants to look out for each other, assessing its function was difficult because it was unmeasurable, unlike physical functions. Additionally, even if older men may not need to be heard while they are still healthy and active, they can become vulnerable when their physical functions start to decline. For these reasons, Interviewees A, B, I, J, and K said that a new program that attracts both men and women and can be assessed for its efficiency was needed.

### 3.2. What Are the Power Relationship Dynamics among the People Involved?

People who are involved in the CBSI programs can be grouped into three categories: (1) local government workers, mainly public nurses, from the local comprehensive care center, (2) the group leader, and (3) participants. The government suggests that the goal of CBSI programs is to nurture older people’s willingness to be independent and provide mutual help to extend healthy longevity. By doing so, it is expected that the social capital and cohesiveness of communities will be enhanced. To begin the program, the local government’s effort to educate and motivate older people was significant. As the local government was already providing medical and nursing care under the long-term care insurance system, the power balance already existed. Older residents misunderstood that the CBSI program was supplied by the local government without any cost.

As the CBSI members already functioned in a territorially connected social relationship and conducted activities together, older participants had social roles in the group. For example, when Interviewee N, a district welfare commissioner, took on a CBSI leadership role, his spouse and close friends became his assistants. Interviewee I explained that in rural areas, mainly farming and fishing communities, a territorial connection was meaningful because members supported each other by sharing information and labor. Therefore, power struggles and conflicts rarely emerged in the group. At the same time, their strong unity became a barrier for potential members to join the group.

Second, the relationship between the local government and older people has changed over time. Older people over 75 years of age tend to have a stronger bond with and faith in the local government, so they are more likely to accept its suggestions. However, the relationship between the local government and older people has changed with technological advancement and agricultural decline. The younger-old, especially those under 70 years, tend to take an individualistic business approach to their work, and are less likely to rely on the local government; consequently, they become careful or critical of government suggestions. They also tend to dislike being constrained by others. Thus, a culture of older people can be age- or cohort-specific. Interviewees A and B from Case 1 were especially concerned about how they could mediate across different age cohorts for the future of their community.

### 3.3. What Are the Benefits and Drawbacks of a Target Social Innovation for People or Society?

Participants in Case 1 CBSI regained grip strength, walking speed, and chair standing ability after attending the health promotion program [28]. We did not have access to information about the long-term influence of the CBSI program for Case 2. Additionally, the participation rate of older people who live alone is high for both Case 1 and Case 2, which shows that CBSI programs help maintain participants’ safety and well-being. One of the female participants of Case 2, who lives alone, said, “My family members live nearby. They come to see me often to make sure I am okay. I am happy to see them, but it is not easy to talk with them because my hearing is not very good. However, I can talk and have many laughs here because we all have similar hearing levels”. Conversations with younger people, including family members, can sometimes be stressful because the elders cannot keep up due to hearing loss. Older people at CBSI meetings can have a stress free-time.

These effects are especially evident for older women. Even before the CBSI program starts, older female participants had a chance to regularly get together at a salon to have a conversation, a cup of tea, and snacks. Although men were entitled to attend the salon, the nature of the activity, sharing conversation, was not attractive to them. According to a male participant of Case 1, Japanese men are accustomed to living based on “work”, “social role”, and “responsibility”, therefore they do not find meaning in meeting and chatting. It seemed that unless men were assigned as group leaders, they would be less likely to find a social role in the group.

Furthermore, older men in rural areas are busy cropping fields, and their work can be physical, so such CBSI programs are not attractive to them. Interviewee I said that local governments should encourage older men to exercise more by providing well-maintained roads for speed-walking and monitoring their distance and speed. Alternatively, they should provide older men chances to enjoy competitive and purposeful activities such as ground golf or mahjong, or activities based on their hobbies and interests, such as fishing or motorcycling. He said that encouraging men to participate in the current form of CBSI programs is “Japan’s issue”.

The public nurses from the local comprehensive care center also experience benefits and drawbacks. Looking at the positive aspects, public nurses have less concern and worry about the health of older individuals because CBSI programs are based on members’ self-help and mutual help. However, it will take some time for them to reach this goal. For example, Interviewees L and M, who are public nurses for Case 2, told us that they are currently responsible for establishing CBSI programs in 6 to 10 communities. To maintain the program’s sustainability, public nurses must thoroughly explain to older people the facts about depopulation, population aging, the social security system, and the decline of the government budget. At the same time, they must understand the human relationship characteristics within each community to provide appropriate emotional support. CBSI programs under the local comprehensive care system rarely succeed without the continuous support of public nurses. This is also a negative consequence of this system, because public nurses must undertake much work.

### 3.4. Does Financial Investment Lead to The Success of a Target Social Innovation?

We observed that the two CBSI groups did not require much financial investment, because they already had necessities such as a public hall, chairs, and whiteboards. The local government rented the necessary materials for exercise, such as videos and/or DVDs and wrist/ankle weights for groups willing to start a CBSI program. Both CBSI groups initially needed to renovate their meeting place to enable barrier-free access and physical exercise, but they did not have to build a place. Interviewee B said, “We did not need much money. Just needed to renovate the meeting place by placing a new heater and wood floors”. Case 2 required a slightly higher budget because the meeting places in their community were very old and unsafe. Interviewees L and M recalled with amusement that their old meeting place was scary because “the floors were tilted and ceilings were about to fall on us”. Other than that, they reused necessary equipment, such as chairs and whiteboards, which the community was already using. The CBSI members occasionally contributed cash for snacks and tea through their generosity.

## 4. Discussion

Using two cases, we introduced the history, development, and role of Japan’s rural CBSI programs. In this section, we discuss the challenges that these programs face and potential ways to improve them. We also talk about how programs in Japan are similar to or different from programs in other countries.

As we explained, the CBSI programs in Japan have the right target to overcome national and community concerns: older people run the health promotion programs for themselves based on self-help and mutual help. People of various ages and with various health statuses are expected to help each other, and participants are given social roles in the groups. For example, younger and healthier older people take the initiative to run the program, and members provide support for the program’s operation in Case 1 and Case 2. Such social interactions help to enhance community cohesiveness. For communities, especially in rural areas, introducing a CBSI program is beneficial because it aims to enhance social capital and cohesiveness. In rural Japan, a collectivistic approach to uniting the community is traditionally common; consequently, older people’s culture regarding CBSI was quickly accepted. By having a chance to meet regularly with the shared purpose of staying healthy, older people can develop a sense of unity and cohesiveness.

Even so, given that the advancement of population aging in the future, the current form of Japan’s CBSI programs faces difficulties. For example, the power balance may be a source of concern. These programs rely on self-help and mutual help among older people of various ages and with various health statuses. Although the idea that younger and healthier older people can assist their peers in promoting health is good, finding a physical exercise effective for all CBSI members is challenging. The members of these two groups currently adjust their physical exercise load by using wrist and ankle weights. Nevertheless, there will be a point at which the current exercise program will no longer effectively enhance some members’ physical strength. Younger and healthier older people in a community may need other types of exercise to maintain their health. Additionally, the current form of CBSI programs does not encourage men’s participation. Thus, forming CBSI programs that target groups based on age, physical strength, and gender may be necessary. By doing so, however, separation within the community would be inevitable. For example, the younger-old, who are less connected to the local government, may withdraw from the program’s activities. Old-old residents, especially those who need physical assistance (e.g., walking), may be transferred to participate in other care programs. When that happens, they will lose contact with the members of the CBSI program.

Another challenge is that CBSI members may need to find ways to increase meeting times for exercise to improve or sustain their physical condition. It is especially evident for programs such as Case 2, when the meeting is hard to operate during wintertime. Besides, the advancement of depopulation and population aging appears inevitable, thus CBSI programs may need to encompass residents from a greater distance. If that is the case, older participants will require transportation, such as a taxi or shuttle bus, otherwise they would not be able to participate. One way to increase meeting times and participation rates is to set up group-specific meetings. A financial investment in developing exercise programs using IT or virtual networking technology could make that happen. This was especially true in 2020, when many older people were self-isolated at home because of the COVID-19 pandemic. The importance of social interaction among older people is an issue, especially in rural areas. Thus, the program budget could be used to develop a system that allows virtual participation.

CBSI programs are promoted to encourage older people to support each other in successful aging. Sharing such a goal, programs have been established and practiced across the world. As we mentioned before, China and Serbia use the foundation style of CBSI. Older people in local communities take the initiative to run programs to meet their needs (e.g., emotional support, access to hospitals). In Ukraine and Lebanon, upper-class older women initiate user-driven educational and training programs for older people. Their programs are mainly self-funded, such as by donations and membership fees. Foundational and user-driven CBSI programs use a bottom-up format that is less connected with the local or national government. On the other hand, the governments in Russia and Vietnam are using the top-down approach to CBSI programs, referred to as state support. The program size is relatively large, and the government targets older people who need support (e.g., due to poverty or sickness). Chile, Sri Lanka, and Iran use the adaptive approach. The size of their CBSI programs is often large, and older participants can talk with members from the government.

Japan’s CBSI programs, which we observed, have the characteristic of being adaptive in that they combine the bottom-up and top-down approaches to promote older people’s well-being. However, Japan’s programs are smaller than those in Chile, Sri Lanka, and Iran, so they are less costly. More precisely, the Japanese government previously made a sizeable financial investment, but then reduced the cost by giving the program initiative to communities. The members of Japan’s CBSI programs have space to be creative, but they are still under specific national/local government guidelines. In that sense, the older members of foundational and user-driven programs may be more critical and have the power to change their situation compared to members of programs with an adaptive approach.

Another form of Japan’s CBSI program is the college of senior citizens in Osaka. It was previously funded by the local government, but the government subsidies were stopped in 2009. Since then, older students took the initiative to reform the system. As a result, it is currently run as a nonprofit organization with almost 3000 students. The approach is bottom-up with the following characteristics of CBSI programs: (1) members enjoy learning with peers; (2) members cherish people’s hobbies; (3) members share hobbies with others; (4) members work with the local government for the community; (5) members try to sustain their health and life goals via activities; and (6) members act on global environmental issues as being the responsibility of older people [29]. The bottom-up approach was possible for them partly because they are located in an urban city with a large population of older people. They can recruit many older people, and members can share roles and responsibilities to sustain the college. However, such a system is hard to operate in rural areas where the population of older people is smaller. Thus, Japan’s CBSI programs took a unique trajectory based on the country’s financial situation, government policies, aging population, and awareness of older people. Aspects of Japanese cases can be used to construct CBSI programs in other countries.

## 5. Conclusions

In this paper, we discuss a social innovation known as CBSI. CBSI programs in Japan emerged under the rapid growth of the aging population and a tight national budget for older people’s medical and nursing expenses. The goal of CBSI programs is to create a system in which older people across a wide age range can help each other sustain their physical and psychological health, and extend their healthy longevity for as long as possible. By doing so, older people can be healthy and happy, and the government can find more efficient ways to use the national budget and human resources.

Based on our two case studies, we found that the current form of Japan’s CBSI programs has benefits and challenges. One positive aspect of these programs, especially in rural areas, is that they unite older people by setting a mutual goal to stay healthy. The nature of collectivist rural culture fits the characteristics of CBSI; however, programs in rural areas also face challenges. For example, the gendered division of labor has been practiced over time; consequently, men and women have different interests and preferred communication styles. In the two case CBSI programs, women tended to find joy and comfort in human relationships and sharing. However, men expressed feelings of discomfort in such situations and instead enjoyed having goal-oriented activities or competition. Therefore, the recruitment of male participants is difficult, and a different approach to this is needed.

The cohort differences in terms of the relationship between older people and the local government are another concern regarding CBSI programs. Old-old people are more likely to rely on and listen to government workers’ suggestions because they grew up in a culture of working with the local government. Conversely, the younger cohort of older people did not have many opportunities to work with local governments under different political policies and business privatization. Thus, older peoples’ diverse attitudes toward CBSI programs that implement government initiatives should be resolved in the future. Regardless of the changing situation, we can conclude that the Japanese style of CBSI programs must continue by implementing modifications. The smiles and laughter of older participants that we witnessed convey everything.

## Figures and Tables

**Table 1:** Staff members.

**Case 1 CBSI**	**Backgrounds**	**Gender**	**Interview Format**
Interviewee A	Stuff member from regional government	Female	Focus group
Interviewee B	Public nurse	Female	Focus group/one-on-one
Interviewee C	Executive director of human resource center	Male	Focus group
Interviewee D	Staff member from human resource center	Female	Focus group
Interviewee E	Staff member from human resource center	Female	Focus group
Interviewee F	Staff member from human resource center	Female	Focus group
Interviewee G	Executive director of CBSI	Male	One-on-one
Interviewee H	Member of CBSI	Female	Focus group
**Case 2 CBSI**			
Interviewee I	Staff member from regional government	Male	Focus group/one-on-one
Interviewee J	Staff member from regional government	Male	Focus group/one-on-one
Interviewee K	Staff member from regional government	Male	Focus group/one-on-one
Interviewee L	Public nurse	Female	One-on-one
Interviewee M	Public nurse	Female	One-on-one
Interviewee N	Executive director of CBSI	Male	Focus group
Interviewee O	Member of CBSI	Female	Focus group

## Data Availability

No new data were created or analyzed in this study. Data sharing is not applicable to this article.

## Supplementary Materials

The following are available online at https://www.mdpi.com/article/10.3390/ijerph18094970/s1, Interview Guides.
