# Supplementary material for: CBSI as a Social Innovation to Promote the Health of Older People in Japan
Source: Int J Environ Res Public Health. 2021 May 7;18(9):4970. doi: 10.3390/ijerph18094970 (PMC8125680; doi:10.3390/ijerph18094970)
Supplement: Supplementary file 1 [file ijerph-18-04970-s001.zip › ijerph-1183194-supplementary.pdf]

Supplementary: Interview Guides

Interview Guide for CBSI staff

Background

1. Please share a brief overview of your organization's work for older people in [*Name of Area*] and how you transitioned into your current role.
2. What is your role in [*Name of CBSI*]?

Resources to support CBSI

3. When was the [*Name of CBSI*] established? Who set it up, and why was it established?
4. What are the overall aims/objectives of the [*Name of CBSI*]? Have these changed over time?
5. How is [*Name of CBSI*] funded? Has this changed over time?
6. Who is involved in the day-to-day activities of [*Name of CBSI*]? What sort of skills do they have? What sort of training and guidance have these persons received?
7. Are you aware of any other similar programs/initiatives to the [*Name of CBSI*] currently underway in [*Name of Area*]?

Engagement with older people and functioning of the CBSI

8. Please describe how older people are engaged in the activities of the [*Name of CBSI*]. Who is eligible to take part in [*Name of CBSI*]?
9. Please can you describe the activities involved in [*Name of CBSI*]?
10. Are you aware of any efforts to monitor/evaluate the [*Name of CBSI*]? How do you know it is achieving what it aims to achieve?
11. How is the [*Name of CBSI*] organized? How has the management of [*Name of CBSI*] evolved?

Outputs/linkage

12. Based on your experience with/knowledge of the [*Name of CBSI*], which factors have helped the program?
13. What about factors that may have hindered the program?
14. How does [*Name of CBSI*] fit with the current health and social care system?
15. How would someone working for [*Name of CBSI*] interact with the other persons working in the health and social care system?

Impact of the CBSI

16. In your experience, is (or can), [*Name of CBSI*] contributing to the health and wellbeing of older people in [*Name of Area*]? In what ways? Is there evidence of this?
17. Where do you see the most significant opportunity for the [*Name of CBSI*] to have an impact in the future? How do you see this being achieved?
18. What advice would you give to people from other place (countries) that would like to implement a similar program in their communities?

Interview Guide for CBSI Leader and Participants

1. Can you tell me how you learned about [*Name of CBSI*]?
2. What made you want to become involved with [*Name of CBSI*]? Why did you want to participate in [*Name of CBSI*]?
3. Are you aware of other similar programs/initiatives to the [*Name of CBSI*]? Have you been involved in any?
4. Can you tell us how you are involved in [*Name of CBSI*]?
5. What type of support or services does the ***[Name of CBSI]*** provide to you?
6. What difference do you think the [*Name of CBSI*] is making in your life?
7. Do you feel it is vital that you continue to have ***[Name of CBSI]*** in your community? Why?
8. Is it something you would recommend to friends?
9. What do you feel works particularly well about ***[Name of CBSI]***?
10. What, if anything, do you think you could be improved?
